# Methods for exploring the plant tree of life

**DOI:** 10.1002/aps3.1039

**Published:** 2018-04-06

**Authors:** Matthew A. Gitzendanner, Ya Yang, Norman J. Wickett, Michael McKain, Jeremy M. Beaulieu

**Affiliations:** ^1^ Department of Biology University of Florida Gainesville Florida 32611 USA; ^2^ Department of Plant and Microbial Biology University of Minnesota St. Paul Minnesota 55108 USA; ^3^ Department of Plant Science Chicago Botanic Garden Glencoe Illinois 60022 USA; ^4^ Plant Biology and Conservation Northwestern University Evanston Illinois 60208 USA; ^5^ Department of Biological Sciences University of Alabama Tuscaloosa Alabama 35487 USA; ^6^ Department of Biological Sciences University of Arkansas Fayetteville Arkansas 72701 USA

The past several years have seen a dramatic growth in the tools and technologies used to acquire the data necessary for phylogenetic reconstruction. Not only have the amount of data and the numbers of taxa included in phylogenetic studies steadily increased over time, but the applications of phylogenies have diversified. The field has rapidly moved from single‐gene analyses to phylogenomic analyses with hundreds of genes from all three genetic compartments within a plant cell. Phylogenetic reconstruction methods have diversified to include gene‐tree and coalescent methods. Furthermore, the use of phylogenetic trees has grown well beyond answering questions of relationships to addressing a wide array of issues including community ecology and genome evolution. This special issue of *Applications in Plant Sciences*, “Methods for Exploring the Plant Tree of Life,” is a companion to the special issue of our sister journal, the *American Journal of Botany*, entitled “Using and Navigating the Plant Tree of Life” (Soltis et al., 2018). The papers in these issues explore the current state of plant phylogenetics, investigating our current understanding of the phylogeny itself, as well as how this phylogeny can be used for downstream analyses and testing of ecological and evolutionary hypotheses.

A number of papers in this issue focus on rapidly advancing sequencing technologies, from reduced representation methods to whole genome sequencing, that are becoming increasingly accessible to a growing audience. Chau et al. (2018) and Vatanparast et al. (2018) provide reviews of different methods for sequence capture, focusing on marker selection. Chau et al. (2018) focus on collecting data to resolve rapid radiations, comparing the efficiency of custom‐designed, taxon‐specific probe sets to more universal sets of probes (i.e., conserved ortholog set [COSII], shared single‐copy nuclear genes [APVO SSC], and pentatricopeptide repeats [PPR]) that are designed to work across a broad array of angiosperms. Chau et al. (2018) find that although targeting taxon‐specific markers may not always be necessary, in their case, the taxon‐specific set of loci did provide better assemblies and a better‐supported phylogeny within *Buddleja* L. (Scrophulariaceae) than those obtained with universal probe sets. For researchers wishing to explore the use of custom‐designed probe sets, Vatanparast et al. (2018) compare three different pipelines for selecting markers for target enrichment from transcriptome data (Hyb‐Seq [Weitemier et al., 2014], MarkerMiner [Chamala et al., 2015], and that of Yang and Smith 2014) and highlight how each performs with regard to different measures. They find that the methods result in final matrices that differ in the average number of parsimony informative sites, data set completeness, and number of loci targeted. These documented differences among the pipelines now allow researchers to select the pipeline most suited to their study system.

Although hybrid enrichment is certainly one popular method of producing libraries for sequencing, McKain et al. (2018) provide an overview of the growing options for phylogenomic studies, including microfluidic PCR, RAD‐seq, target enrichment, transcriptomes, and genome skimming. They also offer guidelines for researchers to determine which approaches may be appropriate for their specific study. Finally, as technologies continue to advance, Li and Harkess (2018) argue that whole genome sequencing will reach a wider audience, obviating the need to employ other methods; they also provide a summary of the current state of sequencing and genome assembly technologies that can guide researchers seeking to take advantage of these developments.

Looking toward the growth in other “‐omics” technologies, several papers provide insights for application of these emerging tools in non‐model plants. Harbert (2018) compares two metagenomics pipelines (Kraken [Wood and Salzberg, 2014] and Centrifuge [Kim et al., 2016]) with MegaBLAST (Zhang et al., 2000) using soil samples for paleovegetation studies to reconstruct ancient plant community composition. Each metagenomics pipeline has its strengths, and both outperform the MegaBLAST tool. Opening metabolomics to non‐model organisms, Sedio et al. (2018) use ultra‐high‐performance liquid chromatography–tandem mass spectrometry (UHPLC‐MS/MS) to fingerprint more than 100,000 metabolites in leaf samples from hundreds of tropical tree species, enabling comparative metabolomics and chemical community ecology. Tovar et al. (2018) describe a cost‐effective Raspberry Pi–based setup for high‐throughput phenomics, allowing low‐cost, rapid acquisition of trait data for eco‐evolutionary studies. Together, these studies point to a growing use of new methods that combine our understanding of the tree of life with historical information, species trait data, and ecological interactions to leverage the tree in increasingly diverse fields.

In contrast to these genomic, metagenomic, and phenomic approaches, the remaining contributions to this issue highlight additional techniques that can be used to obtain valuable information involving the tree of life. Endara et al. (2018) focus on how to effectively glean information from the literature, employing a new natural language processing pipeline to process published species descriptions and extract character matrices from textual information. Their method unlocks and makes available the phenotypic data that are recorded in the literature but that have typically been inaccessible for data analysis or trait mapping. A different approach by Zenil‐Ferguson et al. (2018) focuses on the phylogenetic trees themselves as the source of evidence for chromosome evolution. They provide an R package (chromploid) to reconstruct chromosome evolution across large phylogenies by implementing a likelihood model of chromosome number and ploidy changes across an input tree. These advanced approaches represent only a small sample of new methods to better understand the tree of life, which can be used to answer new questions in fields such as evolution, ecology, and taxonomy.

Overall, these papers present an overview of a field that is thriving—this is an exciting time to be studying the tree of life! Technologies are rapidly evolving to the point where whole genome sequencing is tractable for even individual researchers. Phylogenomic approaches to reconstruct species relationships are also becoming standard. As these tools continue to develop, we are gaining the ability to extend beyond the genome to study metabolites and characterize phenotypes with increasingly robust and rapid methods. Natural language processing can ensure that decades of historical literature can continue to inform and contribute to our knowledge. At the same time, statistical methods allow us to use the trees being generated to investigate fundamental questions ranging from genome evolution and polyploidy to community composition and chemical ecology. These methods as well as future approaches are certain to advance our understanding of the tree of life in years to come.
